# Phenotypic plasticity and a new small molecule are involved in a fungal-bacterial interaction

**DOI:** 10.1038/s41598-021-98474-y

**Published:** 2021-09-28

**Authors:** Andrés Andrade-Domínguez, Abigail Trejo-Hernández, Carmen Vargas-Lagunas, Sergio Encarnación-Guevara

**Affiliations:** 1 Centro de Ciencias Genómicas, Universidad Nacional Autónoma de México, Cuernavaca, Morelos, 62210 México; 2 CAS Biotechnology, Parque Científico y Tecnológico de Morelos, Autopista Mexico/Acapulco km 112, Fracc. Santa Fe, Xochitepec, Morelos, CP 62797 México

**Keywords:** Ecology, Microbiology

## Abstract

Nitrogen-fixing bacteria have been extensively studied in the context of interactions with their host plants; however, little is known about the phenotypic plasticity of these microorganisms in nonmutualistic interactions with other eukaryotes. A dual-species coculture model was developed by using the plant symbiotic bacterium *Rhizobium etli* and the well-studied eukaryote *Saccharomyces cerevisiae* as a tractable system to explore the molecular mechanisms used by *R. etli* in nonmutual interactions. Here, we show that the fungus promotes the growth of the bacterium and that together, these organisms form a mixed biofilm whose biomass is ~ 3 times greater and is more structured than that of either single-species biofilm. We found that these biofilm traits are dependent on a symbiotic plasmid encoding elements involved in the phenotypic plasticity of the bacterium, mitochondrial function and in the production of a yeast-secreted sophoroside. Interestingly, the promoters of 3 genes that are key in plant bacteria-interaction (*nifH, fixA* and *nodA*) were induced when *R. etli* coexists with yeast. These results show that investigating interactions between species that do not naturally coexist is a new approach to discover gene functions and specialized metabolites in model organisms.

## Introduction

In nature, organisms do not exist as solitary entities; in contrast, species establish complex interactions in which they are related directly or through intermediate species. These interactions are dynamic and change along a continuum from antagonism to cooperation^1–4^. The change in the nature of interactions can be gradual or abrupt, thus converting beneficial interactions to antagonistic interactions^5^.

When individuals of different species interact, they can adjust their phenotypes in response to their peers to establish an antagonistic or a mutual interaction^6^. Phenotypic changes in a genotype may be morphological, chemical, or physiological changes or can be developmental behavior modifications^7^.

Despite the importance of interactions between species for biological diversification and organization, we still know little about how these relationships emerge and evolve^8^. Currently, different approaches are being followed to conduct studies on natural interactions. Many of the natural symbioses that we know about are the result of the history of interaction between ancestral populations. The ancestral populations are not available for testing, and we do not know the environmental conditions under which these symbioses were originally established. In contrast, artificial systems allow us to understand the processes that underlie the emergence of a biological interaction and establish causal relationships between environmental and genetic changes^5^.

*Rhizobium* species are gram-negative bacteria that can exist in two states : as free-living saprophytes in the soil and in a symbiotic relationship with leguminous plants. The symbiosis between nitrogen-fixing rhizobia and members of the legume family has emerged and evolved over the past 65 million years^9^. The legume–rhizobium interaction involves a specific molecular signal exchange between the plant and the free-living bacteria, ending with rhizobia eliciting the formation of root nodules. In this interaction, rhizobia supply ammonia or amino acids to the plant and, in return, receive organic acids (principally as the dicarboxylic acids succinate and malate) as a carbon and energy source^10^.

Much of the biological knowledge of rhizobia has been limited to the study of the mechanisms involved in their interactions with their host plants. However, little is known about the biology and phenotypic plasticity of nitrogen-fixing bacteria during their interactions with microorganisms as free-living saprophytes.

Here, we studied the molecular and genetic bases of phenotypic plasticity and ecological dynamics of a fungal–bacterial community. We grew *Saccharomyces cerevisiae* and *Rhizobium etli* on a minimal medium that promoted biofilm formation by both species. We showed that the fungus promotes the growth of the bacteria, and a mixed biofilm is formed whose biomass is ~ 3 times greater and is more structured and stable than that of either single-species biofilm. These biofilm traits are dependent on a plasmid encoding elements involved in bacterial phenotypic plasticity and the production of a novel small molecule secreted by *S. cerevisiae*. Our results show new aspects of the biology of rhizobia during a nonmutual interaction with a unicellular eukaryote. Finally, we showed that investigating ensembles of communities of species that do not naturally coexist (new encounters) may be a new approach to discover gene functions involved in bacterial phenotypic plasticity and the production of specialized metabolites in model organisms.

## Results

### Synergy between *S. cerevisiae* and *R. etli* in biofilm formation

**Figure 1:**
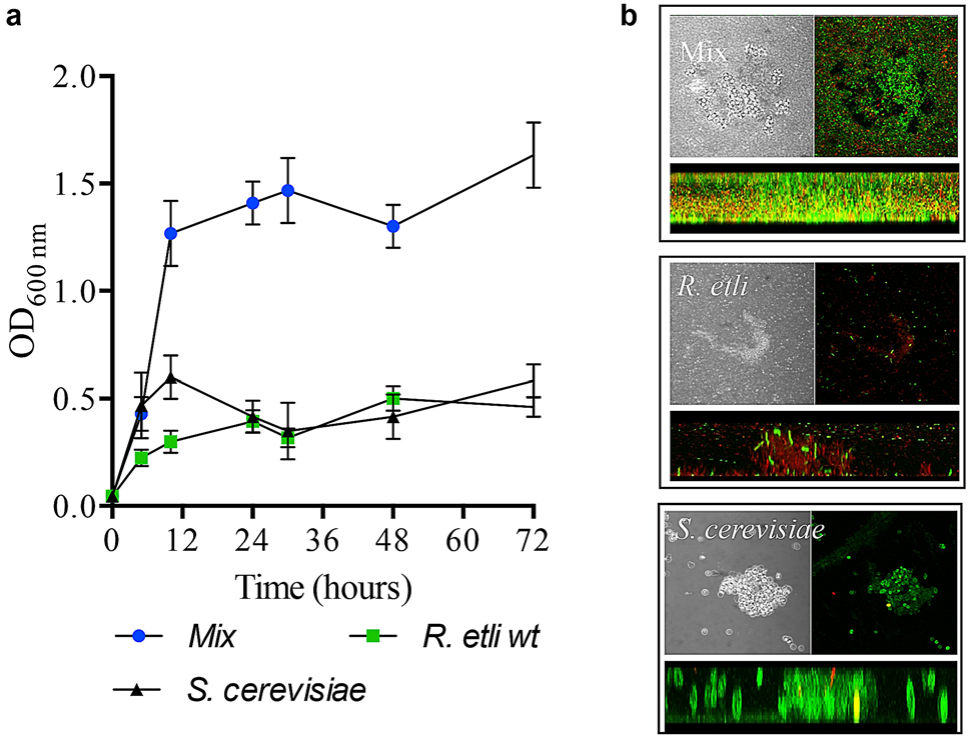
The interaction between *S. cerevisiae* and *Rhizobium etli* CE3 results in the formation of a structurally complex and more productive biofilm in terms of biomass. (**a**) Biofilm formation of *R. etli* CE3 and *S. cerevisiae* Σ1278h Mat α and biofilm growth over time in minimal dextrose medium. The data are representative of 3 independent experiments +/− the S.D. values. (**b**) Top view and cross section of confocal micrographs of the *S. cerevisiae-R. etli* mixed biofilm and the single-species biofilms. Magnification 40 × . The images are representative of 3 independent experiments. Biofilms were developed on glass microscope slides and stained with a LIVE/DEAD viability kit. Red fluorescence indicates dead cells, and live cells are colored green. Images were acquired 24 h after inoculation.

When *S. cerevisiae* Mat α Σ1278h and *R.etli* CE3 were grown in minimal medium with low glucose concentrations (0.1%), these species adhered to abiotic surfaces to form biofilms (Fig. 1). Interestingly, *R. etli* and *S. cerevisiae* formed a mixed biofilm whose biomass was ~ 3 times greater than that of either single-species biofilm (Fig. 1a). In addition, at 24 h, the number of colony-forming units (CFU)/cm2 of *R. etli CE3* in the mixed biofilm was higher than that in the pure biofilm (Supplementary Fig. 1). Confocal laser scanning microscopy of biofilms stained with the Live/Dead Kit (propidium iodide and SYTO9) showed that in the mixed biofilm, the yeast cells formed patches, and the bacterial cells covered most of the surface (Fig. 1b). In contrast, monospecies biofilms of *R. etli* and *S. cerevisiae* had lower structural complexity and contained a greater (80%) number of dead cells, and their individual densities were lower than their populations in the mixed biofilm (Fig. 1b). These results suggest that in mixed biofilms, *S. cerevisiae* promotes bacterial growth.

### *S. cerevisiae* secretes dicarboxylic acids that promote *R. etli* growth and biofilm formation

**Figure 2:**
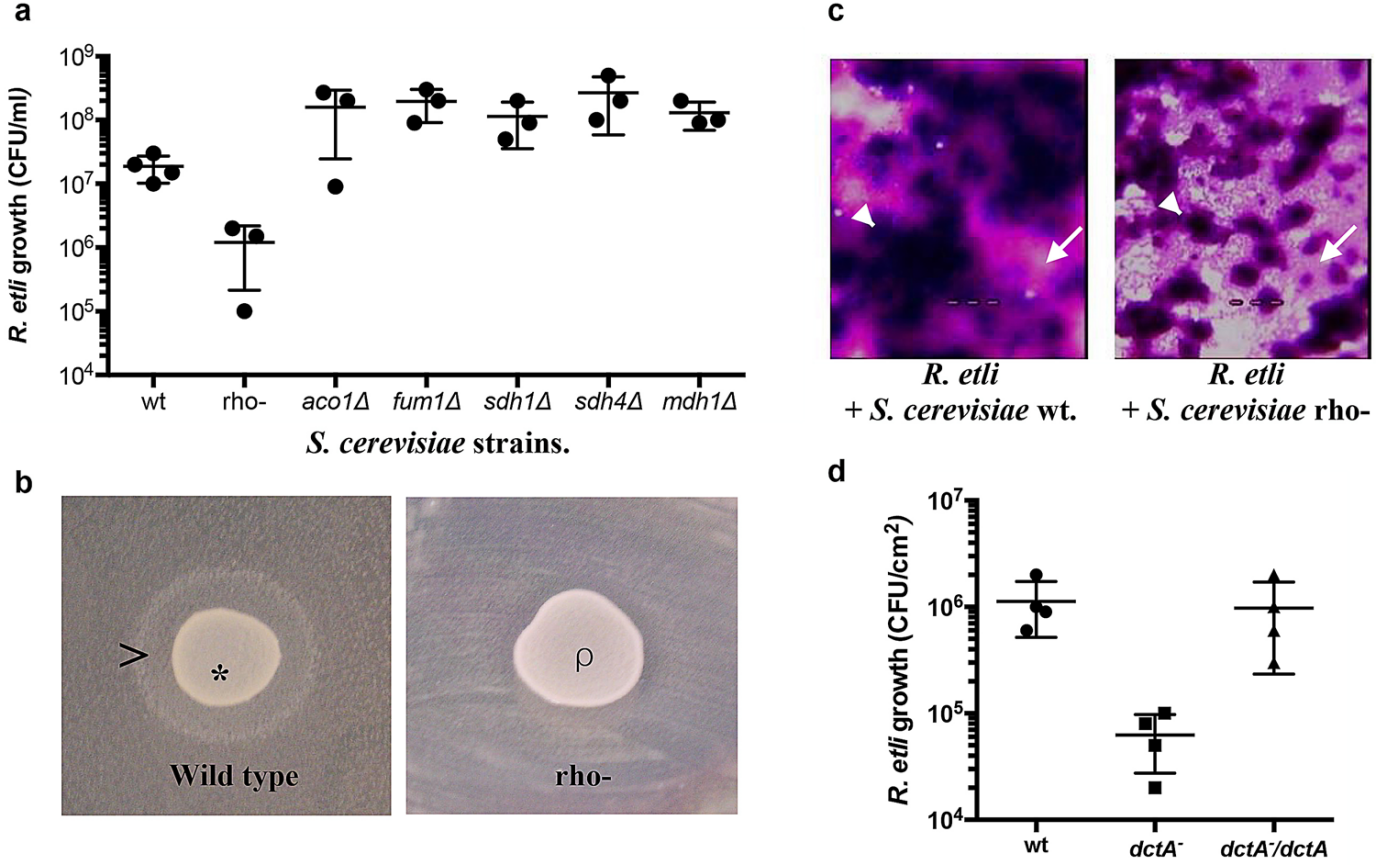
Yeast cells produce dicarboxylic acids that promote the growth of *R. etli*. (**a**) *R. etli* growth in coculture with *S. cerevisiae* BY4741 mutants (*aco1Δ*, *fum1Δ*, *sdh1Δ* and *mdh1Δ*) that accumulate dicarboxylic acids and a BY4741 strain with blockade of the aerobic respiratory chain (rho-). (**b**) Test on solid medium showing that *S. cerevisiae* BY4741 (*) secretes compounds that promote bacterial growth ( >). In contrast, BY4741 rho- cells (ρ), which do not produce dicarboxylic acids, do not promote the growth of *R. etli* CE3. *R. etli* CE3 cells were spread over MMD agar, and yeast cells were spotted in the center. (**c**) Top view of light micrographs of dual-species biofilms; *S. cerevisiae* (arrowhead) and *R. etli* (arrow). Biofilms were developed on glass microscope slides and stained with crystal violet. Magnification 20 × . The images are representative of 3 independent experiments. (**d**) Growth of *R. etli* strains in coculture with *S. cerevisiae* BY4741. The growth of the rhizobium strains was estimated at 24 h. *R. etli* CE3 strains: wild-type (wt), *dctA-* containing an empty expression plasmid (*dctA-*) and *dctA-* containing a plasmid expressing *dctA* (*dctA*-/*dctA*). The data are representative of 3 independent experiments +/− the S.D. values.

We found that the *R. etli* colonies that grew close to *S. cerevisiae* on solid glucose minimal medium were larger than those growing far from yeast colony (Fig. 2).

We used a visual growth promotion assay on solid medium to screen for *S. cerevisiae* knockout strains (YKO library) that influenced bacterial growth. 159 yeast mutants were unable to promote *R. etli* CE3 growth (Supplementary Table 3). In general, these mutants were defective in mitochondrial function. Interestingly, we found that 5 strains with mutations in genes coding for enzymes involved in the TCA cycle showed an enhanced ability to promote bacterial growth compared to that of the wild-type strain (Fig. 2a).

To determine how the *S. cerevisiae* mutants may affect the fungal-bacterial interaction, we analyzed factors that may be altered in mutants with mitochondrial function defects and a compromised TCA cycle.

We compared the production of TCA intermediates between the wild-type and mutant yeast strains. Mutants defective in mitochondrial function (*mef1Δ, gep5Δ, sdh2Δ, ppa2Δ, imp1Δ, cox7Δ, cyc1Δ and cyc2Δ)* produced low amounts of tricarboxylic acids (Supplementary Fig. 2a). In contrast, the aconitase mutant (*aco1Δ*) produced 60% more citrate and succinate; the fumarase mutant (*fum1Δ*) resulted in fumarate accumulation; the succinate dehydrogenase mutants (*sdh1Δ* and *sdh4Δ*) produced 80% more succinate; and the mitochondrial malate dehydrogenase mutant (*mdh1Δ*) produced 60% more malate and succinate (Supplementary Fig. 2b). These results suggested that the large quantities of tricarboxylic acids secreted by the mutant yeast played a role in promoting bacterial growth in the cocultures.

We analyzed the biomass of mixed biofilms formed by yeast cells defective in mitochondrial function (Σ1278B petit mutant). The ability of the wild-type and the petit mutant strains to form a monospecies biofilm was similar (Supplementary Fig. 3). In contrast, the mixed biofilm formed by yeast cells defective in mitochondrial function was significantly lower in biomass than that formed by the wild-type yeast strain (Fig. 2c). Also, Σ1278B petit mutant produced low amounts of tricarboxylic acids (Supplementary Fig. 2a).

We next measured the biomass of the mixed biofilm formed by *S. cerevisiae* and a *Rhizobium* mutant unable to take up C4-dicarboxylic acids (*dctA*-). This evaluation revealed that C4-dicarboxylate uptake by *R. etli* is necessary to form mixed biofilms with high biomass (Fig. 2d).

### A symbiotic plasmid is involved in the phenotypic plasticity of *R. etli.*

**Figure 3:**
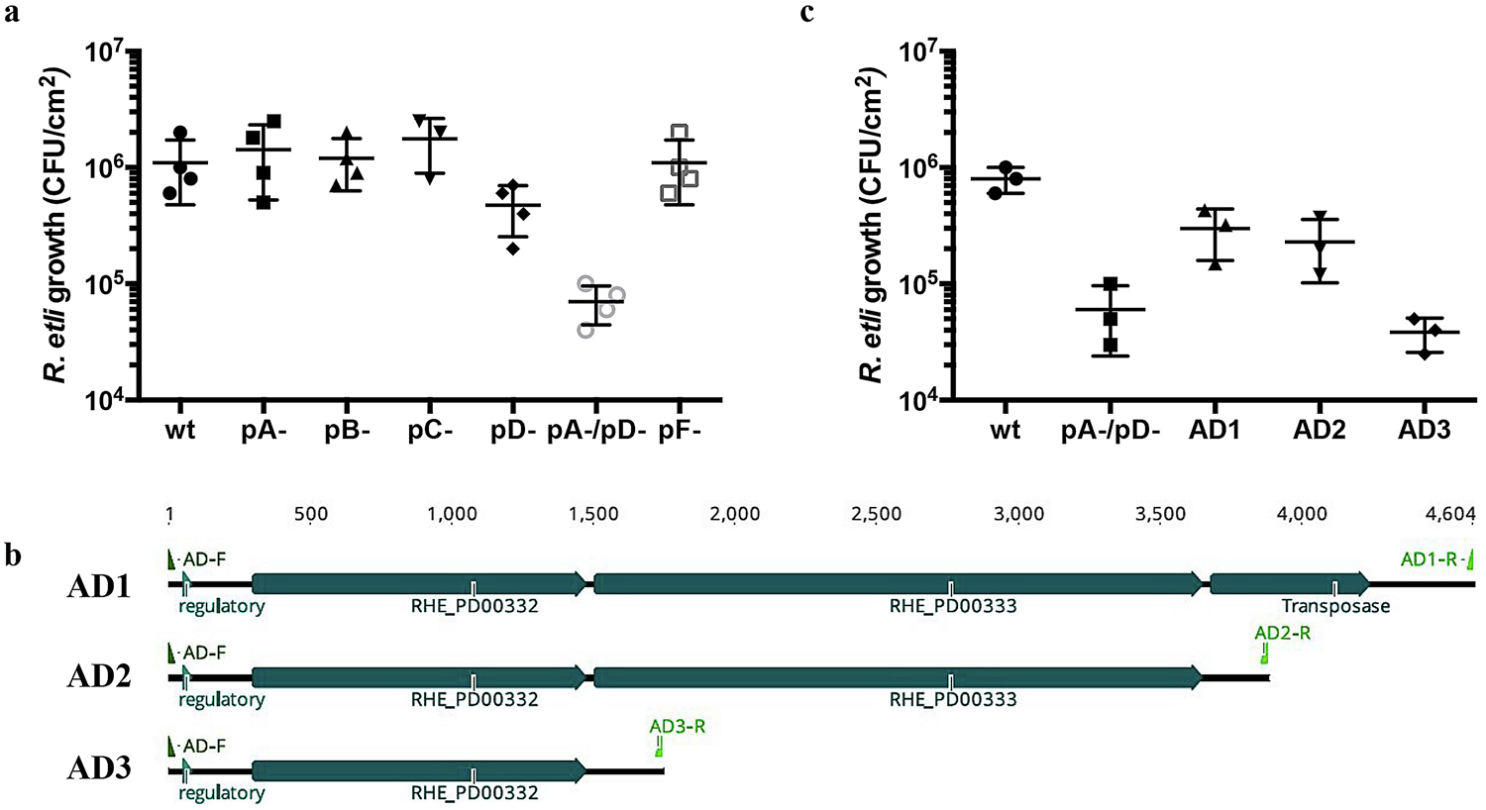
Plasmids pA and pD encode proteins performing functions that are necessary for the coexistence of bacterial cells with yeast. Growth of *R. etli* strains in biofilms with *S. cerevisiae* S1278B. (**a**) Growth in mixed biofilms of *R. etli* strains lacking the plasmids; pA, pB, pC, pF and in one case of two plasmids, pA-/pD-. The growth of the rhizobia strains was assessed at 24 h. (**b**) Scheme of the genes contained in a cosmid that partially complements the growth of the pA-/pD- strain in mixed biofilms. Here, 3, 2 and only one gene was amplified to generate the plasmids AD1, AD2 and AD3, respectively, as indicated in the figure. (**c**) Growth of *R. etli* strains in mixed biofilms. Strains AD1 and AD2 are *R. etli* pA-/pD- cells that carried plasmids AD1 and AD2, respectively. The growth of rhizobium strains in mixed biofilms was estimated at 24 h. The data are representative of 3 independent experiments +/− the S.D. values.

The genome of *Rhizobium etli* CE3 is composed of a chromosome and 6 plasmids (pA, pB, pC, pD, PE and pF)^11^. To determine whether elements encoded by these replicons can participate in the establishment of commensalism, we evaluated the formation of biofilms by yeast and *R. etli* strains lacking these replicons^12^. We found that lack of pA, pB, pC or pF did not affect the ability of bacteria to coexist with yeast (Fig. 3a). Interestingly, a strain cured of plasmids pA-/pD- could not coexist with *S. cerevisiae* to form a mixed biofilm and obtain the benefits provided by the fungus (Fig. 3a).

To determine the genetic elements from the symbiotic plasmid involved in the interaction with yeast, we complemented the *R. etli* pA-/pD- strain with a cosmid library containing fragments of partial digestion (EcoRI) of the *R. etli* CE3 genome^13^. We found that a cosmid containing 9 ORFs from plasmid pD (GenBank: U80928.5) partially restored the ability of *R. etli* pA-/pD- to form a mixed biofilm (Fig. 3b). This cosmid contains 7 insertion sequences (IS) and a predicted operon encoding a probable peptide pheromone/bacteriocin exporter (RHE_PD00332) and a probable bacteriocin/lantibiotic ABC transporter (RHE_PD00333) (Fig. 3b).

The complete operon or only the ABC transporter gene, including its endogenous promoter and terminator regions, was cloned into plasmid pBBR1MCS-3, and the resultant plasmids were named AD1, AD2 and AD32, respectively (Supplementary table 1 and 2). We found that complementation with the complete operon (plasmid AD2) partially restored the ability of *R. etli* pA-/pD- to form a mixed biofilm with yeast (Fig. 3c). In contrast, complementing with the RHE_PD00332 gene (plasmid AD3) does not restore the phenotype. It is necessary to complement only with the RHE_PD00333 gene to determine if its product is involved in the phenotypic plasticity of *R. etli*. These results suggest that the ABC transporter gene (RHE_PD00333) is involved in the fungal-bacteria interaction.

### *S. cerevisiae* produces a small molecule that affects *R. etli* growth

**Figure 4:**
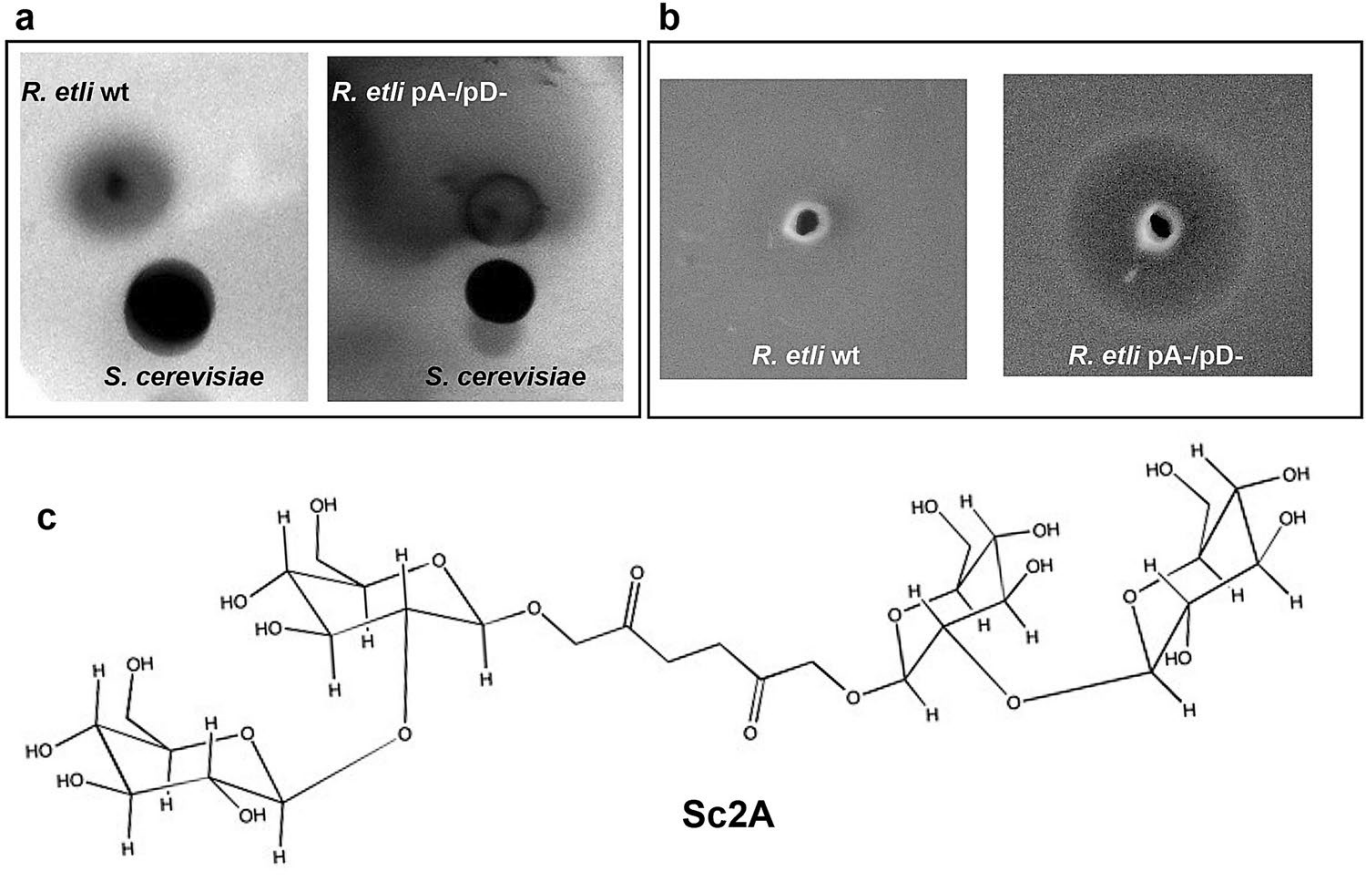
*S. cerevisiae* s1278B produces a small molecule that only affects the growth of *R. etli* strains that do not harbor the symbiotic plasmid and plasmid A. (**a**) *S. cerevisiae* and *R. etli* strains were inoculated in close proximity onto MMD soft agar. *R. etli* pA-/pD- grew, forming a swarm far from the yeast colony. (**b**) Inhibition of *R. etli* pA-/pD- growth by 5 µg/mL of a purified compound from the yeast supernatant, which we named Sc2A. (**c**) Proposed molecular structure of Sc2A.

To determine how *S. cerevisiae* affects the growth of *R. etli* pA-/pD- (Fig. 4a), we evaluated the inhibitory activity of methanol extracts of *S. cerevisiae* culture supernatants.

Interestingly, we found that the methanol extract inhibited *R. etli* pA-/pD- growth but had no activity against wild-type *R. etli* (Fig. 4b). We investigated the chemical constituents of the *S. cerevisiae* culture supernatants. After succesive organic solvent extractions, the methanolic extract was fractionated by HPLC and 8 fractions were obtained. Each fraction was tested for its determine its effect on the growth of *R. etli* pA-/pD-. Only a fraction with the ability to inhibit the growth of *R. etli* pA-/pD- was identified. This resulted in ~ 90% pure sophoroside, judging by its appearance as a dominant peak in the mass spectra obtained by Fast Atom Bombardment Mass Spectroscopy (FAB). As a result, a new sophoroside with bacteriostatic activity, named Sc2A, was isolated (Fig. 4c). The structure of Sc2A was elucidated by a combination of extensive spectroscopic analyses, including 2D NMR and HR-MS.

Sc2A was isolated as a crystalline powder with a positive optical rotation ([α]_D_^25^ + 13.7°, c0.58, H_2_O). The molecular formula of Sc2A was determined to be C_30_H_50_O_24_ from its positive-mode FAB data (m/z 794.26 [M + H]^+^), which was consistent with the ^13^C NMR data. RMN^1^H (CD_3_OD, 400 MHz) data for Sc2A: δ 5.1 d (*J* = 3.6 Hz), 4.4 d (*J* = 8 Hz), 4.23 dd (*J* = 9, 4.8 Hz), 3.79 t (*J* = 10.8, 14.4 Hz), 3.73 m, 3.67 m, 3.639 m, 3.63 dd (*J* = 8, 9.2 Hz), 3.53 dd (*J* = 5.6, 5.2 Hz), 3.36 dd (*J* = 3.6, 4 Hz), 3.31 dd (*J* = 8, 8 Hz), 3.10 dd (*J* = 8, 7.6 Hz), 2.77 dd (*J* = 4.4, 6.8 Hz), 2.61 m, 2.46 m, 2.33 m, 2.12 m. RMN^13^C-DEPT (CD_3_OD, 400 MHz) data for Sc2A: δ 181.2 (C), 175.9 (C), 98.1(CH), 93.8 (CH), 78.05 (CH), 78.02 (CH), 76.30 (CH), 74.92 (CH), 73.80 (CH), 73.11 (CH), 71.78 (CH), 71.72 (CH), 64.37 (CH_2_), 62.87 (CH_2_),62.72 (CH_2_), 57.24 (CH), 30.70 (CH_2_), 26.19 (CH_2_), 28.21 (CH_2_).

The IR spectrum of Sc2A displayed characteristic absorptions of 3416.34 cm^-1^ (O–H), 1642.10 (C = O), 1405.44 (C–OH), 1242.93 (C–O–C), 1040.36 (C-H), and 598.48 (O-C-O).

Sc2A possesses a sophorose linked by 2,5 hexanedione to another molecule of sophorose (Fig. 4c).

### Sc2A induces the expression of genes involved in symbiosis

**Figure 5:**
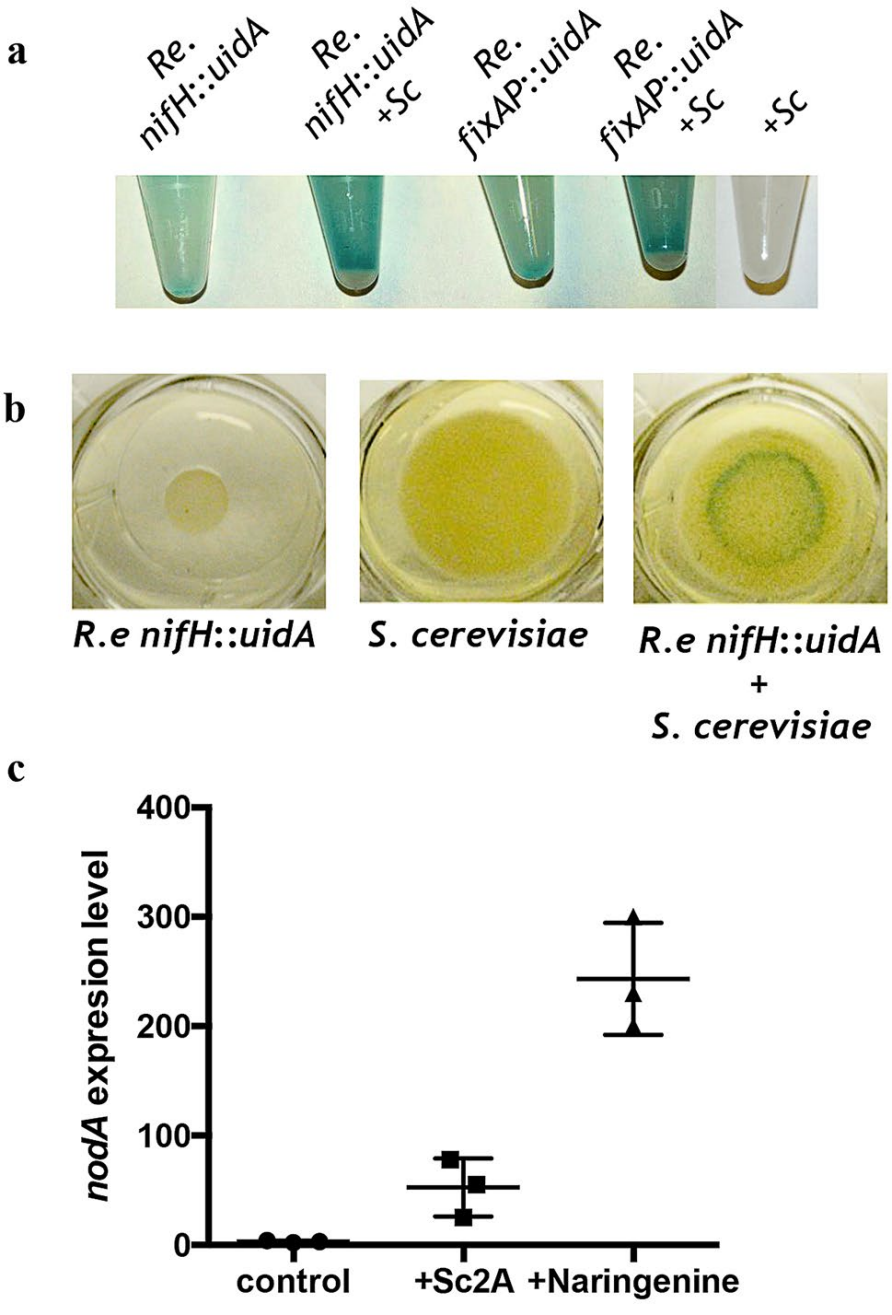
The expression of *Rhizobium etli* genes involved in symbiosis is induced in cocultures with yeast or by exposure to the small molecule Sc2A. (**a**) Activity of different *R. etli* promoters in monoculture (Re) or in coculture with yeast (+ Sc). Cells were cultured for 24 h in 1 ml of PY-D in 1.5-mL tubes. The tubes were kept closed to generate an environment with a low oxygen concentration. (**b**) Activity of the *nifH* promoter in *R. etli* cells grown alone (Re) or in coculture with yeast (+ Sc) on PY-D agar. (**c**) Effect of Sc2A on the expression of the *nodA* gene in *R. etli* cells grown in liquid culture. Cells stimulated with the flavonoid naringenin were included as a positive induction control. The data are representative of 3 independent experiments +/− the S.D. values.

Expression from the *nifH* and *fixA* promoters was studied in *R. etli* monocultures and cocultures with yeast by monitoring GUS activity in living cells. Cells were grown on solid PY-D medium for 1 day, and monitoring of GUS expression showed that the *nifH* promoter was strongly induced when *R. etli* was grown with yeast in liquid medium and on solid medium (Fig. 5).

At the beginning of the symbiosis, the legume roots exude flavonoids, which induces in *R. etli* the expression of a group of genes (*nod*) involved in the synthesis of lipochitooligosaccharides, also called nodulation factors (NFs). Recognition of NFs by the host plant triggers both rhizobial infection and initiation of nodule organogenesis^14^. NodA protein is involved in N-acylation of the chitooligosaccharide backbone of NFs. Given the participation of *nodA* in the interaction of *R. etli* with a eukaryote, we decided to evaluate the expression of this gene in response to exposure to 5 µg/mL of Sc2A (this concentration is similar to that found in cocultures). We found that Sc2A induces the expression of *nodA* (Fig. 5c). However, the levels of induction of *nodA* were moderated compared to the values obtained upon naringenin induction (Fig. 5c).

## Discussion

For thousands of years, interaction with legumes has shaped and directed the evolution of nitrogen-fixing bacteria^10^. However, these bacteria have also been subject to the pressures imposed by the environment and biotic interactions when they live as saprophytes in the soil. As saprophytes, Rhizobium strains compete for resources, communicate, and establish alliances with other soil microbes.

We showed that coculturing *R. etli* and *S. cerevisiae* often leads to increased biofilm formation (Fig. 1). We found that this increase in the mixed biofilm biomass should not be interpreted as simply the sum of the biomasses of the two monospecies biofilms. Colony-forming unit analysis showed that *R. etli* fitness is largely facilitated by yeast in mixed biofilms (Figs. 2 and 3).

Recently, it has been shown that biofilm formation is stimulated as a response to ecological competition in pairwise mixtures of bacterial isolates^15^. In contrast, Ren et al.^16^ observed a high prevalence of synergy in biofilm formation in multispecies consortia isolated from soil. The authors suggested that collective cooperation increases biofilm formation.

Fungi are abundant organisms that inhabit the soil and the rhizosphere. However, little is known about the molecular mechanisms used by Rhizobium species to compete or establish alliances with fungal cells. Our results suggest that rhizobia take advantage of fungal primary metabolism to establish commensal relationships. This hypothesis is supported by the following results: I) *S. cerevisiae* cells that excreted low quantities of dicarboxylic acids were unable to promote *R. etli* growth and biofilm formation. II) Compared with the wild-type strain, *R. etli* mutants unable to take up dicarboxylates were not stimulated by yeast and had low fitness in mixed biofilms. These results suggest that synergy in biofilm formation should be interpreted to be a result of a commensal interaction. To our knowledge, this is the first study to show how a nitrogen-fixing bacterium establishes a commensal interaction with a unicellular eukaryote.

Attachment and biofilm formation on roots are a key process for the subsequent entry of rhizobia into a plant and the development of an effective symbiosis between rhizobia and their host plants^16^. Plants secrete organic acids that attract bacterial cells and promote biofilm formation on the root surface^17^. Interestingly, *R. etli* mutants unable to take up dicarboxylates did not have the ability to establish mutualism with the plant. This inability suggests that transport of dicarboxylic acids not only is an important mechanism of *R. etli* in symbiosis with its host plant but also is relevant in the interaction of *R. etli* with soil microorganisms such as fungi.

A key factor affecting the fitness of an individual is the ability to change its chemistry, physiology, development, morphology, or behavior in response to environmental cues (phenotypic plasticity)^6^. We know little about how the genetic elements involved in mutualism with plants, such as symbiotic plasmids, are involved in interactions with unicellular eukaryotes such as fungi. Here, we explored the role of plasmids in the phenotypic plasticity of *R. etli* during its interaction with a unicellular eukaryote in a structured community (mixed biofilm). The *R. etli* CE3 genome is organized into a chromosome and six plasmids^11^. The symbiotic plasmid, or plasmid pD, contains the most important genetic elements that allow these bacteria to change their physiology during the transition from free-living saprophytes to nitrogen-fixing endosymbionts^11^. We were interested in identifying the genetic elements encoded in the symbiotic plasmid and the other replicons that are involved in the phenotypic plasticity of *R. etli* in response to interaction with *S. cerevisiae*. Interestingly, we found that the *R. etli* strain lacking the pA and pD plasmids was unable to grow and form biofilms with yeast. We identified a putative bacteriocin/lantibiotic ABC transporter (RHE_PD00333) encoded in the symbiotic plasmid that partially complemented the pA-/pD- strain for growth with *S. cerevisiae*. The partial complementation with RHE_PD00333 suggests that other elements are encoded in plasmid pA or pD to generate a complete mechanism to resist the pressures imposed by yeast. Considering our results and the in silico analyzes of the proteins encoded by RHE_PD00332 and RHE_PD00333 genes, we hypothesized that these genes could constitute a system involved in the detoxification of sophoroside Sc2A.

Interestingly, the promoters of 2 genes that are key in plant bacteria-interaction (*nifH* and *fixA*) were induced when *R. etli* coexists with yeast in liquid cultures or when it forms mixed colonies on solid medium plates. Previous work suggests that the expression of these genes only occurs under microarobic conditions within the nodules^18^. However, *nifH* expression has been reported in soil microbial communities. To our knowledge, the regulation of the fix gene expression of *R. etli* when this bacterium coexists with other microorganisms has not been studied. However, we do not know the signaling pathways and the causes of induction of these genes during the fungus-bacteria interaction. We hypothesize that the structural complexity of the mixed biofilm and the high density of cells in it, generates a hypoxic environment that triggers the expression of NifH. Consistent with this, previous research has shown that *nifH* and *fixA* promoters were activated under both free-living microaerobic, and symbiotic conditions^18^.

Interestingly, we found that the soforoside Sc2A induces the expression of the *R. etli nodA* gene. This gene is activated when bacteria senses the flavonoids produced by the plant and the nodulation process begins^14^. NodA expression is not only triggered by the induction of flavonoids, but also by acidity or high salt concentrations^19,20^. Furthermore, del Cerro et al.^21^ found that the non-metabolizable sugar dulcitol also induce the nod genes activation in *R. tropici*. This suggests that the stress caused by Sc2A, or its molecular structure could be the cause of the induction of *nodA* expression. Studies are needed to find out if there is a direct role for the protein NodA in the adaptation of the bacteria to stressful conditions.

A global analysis of the expression of the symbiotic plasmid genes is required to understand the regulation and participation of these genes in *R. etl*i during the establishment of interactions with other organisms outside the nodule of the host plant.

Sophorosides are derivatives of the disaccharide sophorosa, which have been isolated from plants and yeasts. Some plants produce sophoraflavonolosides, which result from the union of a flavonoids with a molecule of sophorosa^22^. Another group of sophorosides with important biological activities are the sophorolipids^22,23^. SLs are a group of extracellular biosurfactants whit antimicrobial activity produced by yeasts associated whit insects and plants (*Candida spp*., *Starmerella bombicola Wickerhamiella domercqiae* and *Rhodotorula bogoriensis*)^22,23^. Here, we report the molecular structure and ecological role of a new sophoroside produced by *S. cerevisiae*. Like the sophorosides produced by other yeasts, Sc2A has antimicrobial activity and therefore can be an important molecule for the establishment and dynamics of yeast-associated microbial communities.

Species belonging to the *Saccharomycetaceae* family are abundant in the rhizosphere and influence plants physiology^24^. However, the ecological roles of yeasts in establishing the microbial communities of the rhizosphere are unknown. Sarabia et al*.*, found that 6 species of yeasts (*Candida sp.* and *Meyerozyma sp.*) are the most abundant in the rhizosphere of maize^25^. These authors also estimated that there is a population of ~ 10^6^ yeasts cells per gram of soil. Interestingly, two species belonging to the genus *Candida* produce sophorosides with antimicrobial activity against *Bacillus subtilis* and *Escherichia coli*^26^. We hypothesized that the RHE_PD00332 and RHE_PD00333 genes of the symbiotic plasmid are key factors that allow *Rhizobium etli* to coexist and benefit from the carboxylic acids secreted by the rhizosphere-inhabiting soforoside-producing yeast.

Taken together, our data and those of other authors suggest that the plasmid that contains most of the genes involved in plant-bacteria symbiosis, also encodes elements involved in the phenotypic plasticity shown by *R. etli* during its integration into yeast communities in the rhizosphere. Understanding the phenotypic plasticity of rhizobia during the interaction whit natural partners (soil microbes) and in artificial communities, can be used to discover new genes and learn about new gene characteristics that have been extensively studied during nitrogen fixation. In addition, this knowledge can be useful for the development of biofertilizers and for the creation of strains that ensure productivity in the crop and reduce the need for artificial fertilizers that are expensive and cause environmental contamination.

Our discovery of a new molecule produced by yeast, shows that the creation of synthetic communities with strains lacking one or more plasmid can be a useful approach for the discovery of specialized metabolites and for understanding the assembly of microbial communities.

## Materials and methods

### Strains, plasmids and culture conditions

The yeast, bacterial strains and plasmids used in the study are listed in Supplementary Table S1 and S2. *R. etli* was grown at 30 °C in PY (0.5% peptone, 0.3% yeast extract and 7 mm CaCl_2_) medium. *S. cerevisiae* was routinely grown in YPD (1% yeast extract, 2% bacto–peptone and 2% dextrose) medium at 30 °C. *Escherichia coli* was grown at 37 °C in Luria–Bertani medium. Antibiotics at the following concentrations were added to each medium to maintain selection for plasmids or to select recombinant strains: nalidixic acid (20 μg/mL), streptomycin (200 μg/mL), tetracycline (5–10 μg/mL), spectinomycin (200 μg/mL), kanamycin (30 μg/mL) and gentamicin (30 μg/mL). Triparental conjugations of *R. etli* were performed as described previously^27^. DNA preparation and recombinant DNA techniques were performed according to standard procedures^28^.

### Static biofilm assays

Exponential phase *R. etli* or *S. cerevisiae* cells were inoculated in 96-well PVC plates (Becton Dickinson, #353,911, conical bottom) in 150 μL of minimal dextrose medium (1.6 Mm KH_2_PO_4_, 0.83 mM MgSO_4_, 10 mM NH_4_Cl, 5 mM CaCl_2_, 1.84 µM FeCl_3_•6H_2_O, 5.55 mM dextrose, 2 µg/mL biotin and 20 μg/mL uracil). To generate mixed biofilms, we inoculated ~ 2 × 10^6^ bacterial cells per milliliter and ~ 2 × 10^5^ yeast cells per milliliter at the start of each experiment. For the single-species biofilms, the medium initially contained ~ 4 × 10^6^ bacterial cells or ~ 2 × 10^5^ yeast cells per milliliter. After incubation at 30 °C, biofilms in the wells were stained for 15 min with 150 μL of 1% crystal violet and washed four times with sterile ddH2O. The crystal violet was dissolved in 150 μL of 95% ethanol, and the OD was measured at 595 nm.

### Determination of population dynamics

The biofilm population of each species in the cultures was determined by calculating the number of CFU (colony-forming units)/cm2. Biofilms were diluted in Tween solution (0.01% Tween 80 and 10 mM MgSO_4_), sonicated for 30 s and plated on PY agar to determine the population densities of *R. etli*. To estimate the *S. cerevisiae* population, biofilms were deflocculated using 300 mm EDTA before being diluted, sonicated for 30 s and plated on YPD agar, as previously described by Smukalla et al*.*^29^.

### Confocal laser scanning microscopy

Biofilms formed on glass slides were analyzed via confocal laser scanning microscopy. After biofilm formation, slides were removed, transferred to 50-ml conical tubes and washed once with fresh medium. To monitor the viability of cells, biofilms were stained using a BacLight LIVE/DEAD staining system according to the manufacturer’s protocol (#L7007, Molecular Probes). Biofilms were stained for 30 min, washed once with fresh medium and observed with a Zeiss LSM 510 META confocal laser scanning microscope equipped with 488-nm, 514-nm, 543-nm and 633-nm laser sources.

### Yeast deletion screening

Yeast knockout strains (YKO library, Open Biosystems, Huntsville, AL, USA) were tested against *R. etli* pd-/pa- to identify the genes involved in the stimulation of *R. etli* growth. The YKO library contained ~ 4700 nonessential gene-deletion mutants. Individual deletion strains (4 μL) were transferred from frozen stocks to YPD (containing 200 μg/mL G418) medium plates using a prong and were grown at 30 °C for 2 days. Then, 96 mutants were spotted (with a 1.5-cm distance between strains) on 24 × 24 cm minimal dextrose agar plates inoculated with *R. etli* CE3. To allow yeast growth, the minimal medium was supplemented with 25 mM dextrose, 20 µg/mL uracil, 2 µg/mL biotin, 20 µg/mL histidine, 100 µg/mL leucine and 20 µg/ml methionine. The plates were incubated at 30 °C for 24 h, and the presence or absence of a growth promotion zone was monitored visually. The mutants that did not promote bacterial growth were retested, and organic acid production was quantified by HPLC as described previously^5^.

### Purification of antimicrobial molecules from *S. cerevisiae* supernatants

*S. cerevisiae* Mat α Σ1278h cells were inoculated in 1 L of minimal dextrose medium and incubated at 30 °C for 1 day. The cells were removed by centrifugation, and the supernatant was filtered with a membrane filters with a pore size of 0.45 µm. Then, the supernatant was lyophilized and soaked in methanol overnight to extract organic components. The methanol extracts were concentrated to dryness in a vacuum. The powder was soaked in a 4/1 acetonitrile/methanol solution overnight. Then, the acetonitrile extract was concentrated to dryness in a vacuum. The powder was dissolved in water and fractionated by reversed-phase high performance liquid chromatography (HPLC). HPLC was performed using a Hypersil 10u C18 preparative column (Hypersil 10 C18, P#0022, 250 × 10.00 mm) at 1 mL/min with a diode array detector. The mobile phase was a linear gradient eluent of acetonitrile–water. The gradient solvent elution profile was as follows: 10–100% acetonitrile for an additional 30 min, 100% acetonitrile for 10 min, and then 10% acetonitrile until the end of fractionation. Eight fractions were obtained, concentrated to dryness and dissolved in water. The fraction collected at 11.3 min was the active in the inhibition of *R. etli* pd-/pa-. The overall yield of the pure compound (isolated as a crystalline solid) was 9 mg/L of culture.

### General chemical analysis procedures

Infrared absorbance spectra were collected with a Fourier transform infrared (FTIR) spectrometer (NICOLET 6700). Positive FAB-MS spectra were recorded on a JEOL MStation JMS700 mass spectrometer using *m*-nitrobenzyl alcohol as the matrix. 1H, gCOSY, and HSQC NMR experiments were performed with a Varian Mercury 400 MHz spectrometer. 13C experiments were performed with a Varian 400 MHz spectrometer equipped with a Varian OneNMR probe. Chemical shifts were referenced to the residual solvent peaks in CD3OD. Optical rotation was measured with a Jasco DIP 360 polarimeter fitted with a microcell (10 mm path length).

### Quantification gene expression in *R. etli*

β-Glucuronidase (GUS) reporter strains were generated by transformation with fusion transcripts containing a specific *R. etli* promoter fused to the *uidA* gene in the broad-host plasmid pBBRMCS53^14^. Quantitative β-glucuronidase activity was determined in 1 mL culture samples using p-nitrophenyl-β-D-glucuronide (PNPG) as the substrate, as described previously^30^. Values were normalized to the total cell protein concentration as determined by the Lowry method. Specific activity values are reported in nM of product per minute per milligram of protein.

### In situ GUS assays

5-Bromo-4-chloro-3-indoxyl-beta-D-glucuronide cyclohexyl ammonium salt (X-GLUC) (Gold Biotechnology, Catalog # G1281C) was used to detect GUS activity in living cells. After 24 h of growth, 0.5 mg/mL X-GLUC was added to the cultures to detect GUS activity. Mixed colonies of *R. etli-S. cerevisiae*, and single species colonies were grown on 1 mL of PY-agar (supplemented with 0.2% dextrose) in 24-well plates. Colonies of a single species were generated by inoculating on the medium 10 µL of a cell suspension containing 1 × 10^6^ CFU/mL of *R. etli* or 1 × 10^5^ CFU/mL of *S. cerevisiae*. Mixed colonies of *R. etli-S. cerevisiae* were generated by inoculating on the agar 10 µL of a cell suspension containing 2 × 10^6^ CFU / mL of *R. etli* and 2 × 10^5^ CFU / mL of *S. cerevisiae*. The plates were sealed with parafilm and incubated for 72 h at 30 C. Afterwards, the colonies were covered with 100 µl of X-Gluc 1 mg/mL in PBS.

### Statistical analysis

All data were calculated as the mean ± s.d. values of data from at least three experiments. Statistical analysis was performed using Student’s *t*-test, and differences between the experimental values and the control values were considered statistically significant at *P* < 0·05.

## Supplementary Information

Supplementary Information.
