# Supplementary material for: Phenotypic plasticity and a new small molecule are involved in a fungal-bacterial interaction
Source: Sci Rep. 2021 Sep 28;11:19219. doi: 10.1038/s41598-021-98474-y (PMC8479133; doi:10.1038/s41598-021-98474-y)
Supplement: Supplementary file 1 — Supplementary Information. [file 41598_2021_98474_MOESM1_ESM.pdf]

Phenotypic plasticity and a new small molecule are involved in a fungal-bacterial interaction.

Authors: Andrés Andrade-Domínguez[1,2], Abigail Trejo-Hernández[1,2], Carmen Vargas-Lagunas[1], Sergio Encarnación-Guevara[1]*

Supplementary Information

Supplementary Figures.

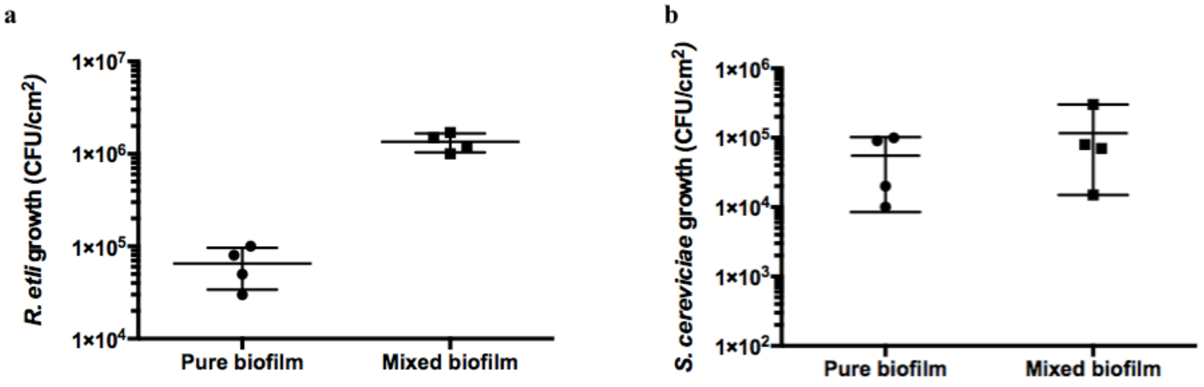

Supplementary Figure S1. a) Growth of *R. etli CE3* in pure biofilm or in mixed biofilm. **b**) Growth of *S. cereviciae* in pure biofilm or in coculture with *R. etli* CE3. The growth was estimated at 24 hours in minimal dextrose medium. The data are representative of 4 independent experiments +/- the S.D. values.

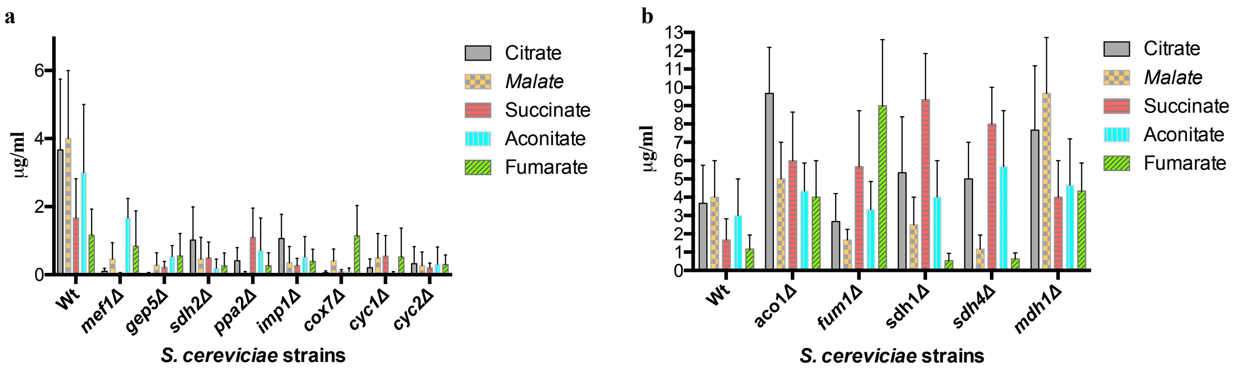

Supplementary Figure S2. C4-dicarboxylates concentrations in the supernatants of *S. cereviciae* mutants (BY4741 background) at 12 hours in minimal dextrose medium. **a**) The graph shows the concentration of organic acids in the supernatant of yeast mutant cultures that do not promote the growth of *R. etli* CE3. Graph **b** corresponds to yeast mutants that accumulate a higher concentration of organic acids with respect to the wild strain (wt). The data are representative of 3 independent experiments ±S. E. M.

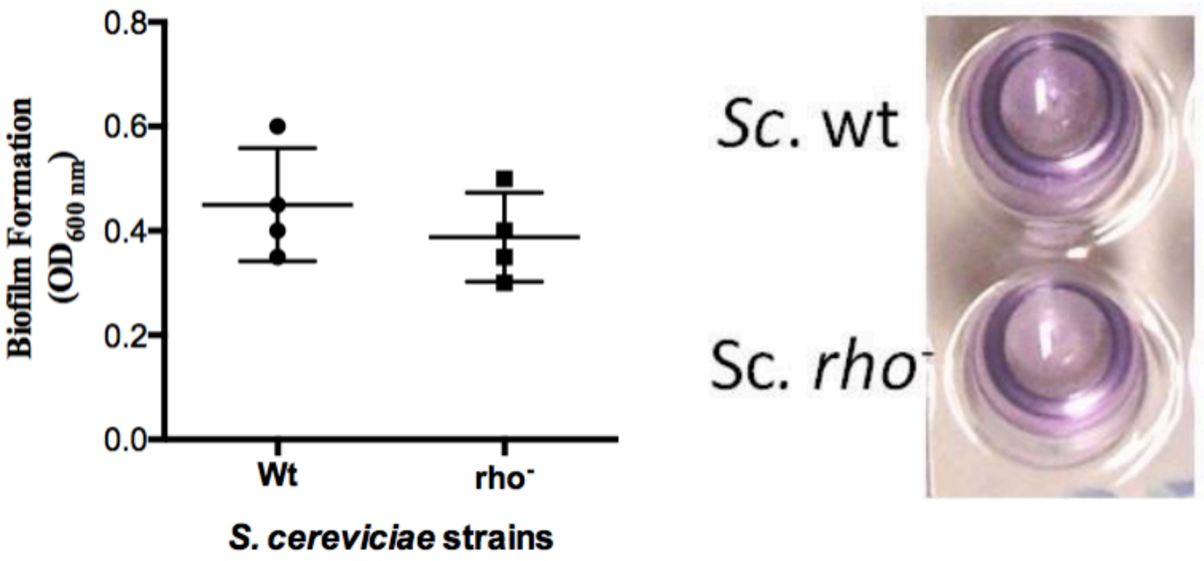

Supplementary Figure S3. Biofilm formation of *S. cerevisiae* Σ1278h Mat α in minimal dextrose medium. Biofilms were developed on PVC plates and stained with crystal violet. Images were acquired 24 h after inoculation. The data are representative of 3 independent experiments +/- the S.D. values.

Supplementary Tables S1-S3

Supplementary Table S1. List of strains and plasmids used in this study.

Strain	Relevant genotype	Reference
R. etli		
CE3	Sm^r derivative of CFN42 strain	Noel *et al*., 1984
pA-	Derivative of CE3 strain, cured of plasmid A.	Brom *et al*., 1992
pB-	Derivative of CE3 strain, cured of plasmid B.	Brom *et al*., 1992
pC-	Derivative of CE3 strain, cured of plasmid C.	Brom *et al*., 1992
pD-	Derivative of CE3 strain, cured of plasmid D.	Brom *et al*., 1992
pE-	Derivative of CE3 strain, cured of plasmid E.	Brom *et al*., 1992
pA-/pD-	Derivative of pD-, cured of plasmid A and D.	This study
CE3*dctA*$^-$	CE3 dctA::ΩKm	This study
E. coli		
DH5α	*supE 44*Δ *lacU169* (φ80*lacZ*ΔM15) *hsdR17 recA1 endA1 gyrA96 thi-1 relA1*	Hanahan et al., 1991
HB101	*supE44 hsdS20* ($r_B^- m_B^-$) *recA13 ara-14 proA2 lacY1 galK2 rpsL20 xyl-5 mtl-1*	Boyer & Roulland-Dussoix D, 1969
S. cerevisiae		
Σ1278h	*MATa, ura3-52*	G. Fink, Cambridge, MA, USA
Σ1278h rho-	*MATa, ura3-52. Lacks mitochondrial DNA, respiration- deficient mutant*	This study
Yeast knockouts strain collection (YKO)	BY4741 background and have a genotype of *his3Δ1 leu2Δ0 met15Δ0 ura3Δ0, Kan*^{r}	Open Biosystems, Huntsville, AL, *USA*
**Plasmids**		
pBBR1MCS5	pBBR1MCS derivative, Gm^r	Kovach *et al.*, 1995
pAD1	pBBR1MCS5 derivative with *RHE_PD00332, RHE_PD00333 and RHE_PD00334* gene (4604 bp) BamHI/XbaI fragment;Gm^r	This study

pAD2	pBBR1MCS5 derivative with *RHE_PD00332 and RHE_PD00333* gene (3879 bp) BamHI/XbaI fragment;Gm^r	This study
pAD3	pBBR1MCS5 derivative with *RHE_PD00332* gene (1740 bp) BamHI/XbaI fragment;Gm^r	This study

Sm^r, streptomycin-resistant; Km^r, kanamycin-resistant; Tc^r, tetracycline-resistant; Sp^r, spectinomycin-resistant.

Supplementary Table S2. List of primers used to amplify RHE_PD00332, RHE_PD00333 and RHE_PD00334 and to construct the vectors pAD.

**Name**	**[a]Sequence 5' > 3'**	**Position 5'>3'**	**Restrict-ion site**
AD1-BamHI-F	TATTGGTACC*GCAGCCATTGAATAA CATAC	346611-346630	KpnI
AD1-XbaI-R	TGAGTCTAGA*TGAAACGGCTCATAT AGG	351197-351214	XbaI
AD2-XbaI-R	TCAAC TCTAGA*GCCTCTATAGCTATGACGTG	350470-350489	XbaI
AD3-XbaI-R	ATCAT TCTAG*CTTGGGGATCTTGTACAG	348333-348350	XbaI

a Underlined, built-in restriction site; asterisk, start of the corresponding position in the *Rhizobium etli* CFN42 genome sequence (GenBank: CP000133.1).

Supplementary Table S3. Yeast mutants that do not promote *R. etli* CE3 growth.

ORF name	GENE	FUNTION
YLR139C	SLS1	Mitochondrial membrane protein that coordinates expression of mitochondrially-encoded genes; may facilitate delivery of mRNA to membrane-bound translation machinery

ORF name	GENE	FUNTION
YLR069C	MEF1	Mitochondrial elongation factor involved in translational elongation
YLR091W	GEP5	Protein of unknown function; detected in highly purified mitochondria in high-throughput studies; null mutant has decreased levels of cardiolipin and phosphatidylethanolamine; not an esssential gene
YLR025W	SNF7	One of four subunits of the endosomal sorting complex required for transport III (ESCRT-III); involved in the sorting of transmembrane proteins into the multivesicular body (MVB) pathway; recruited from the cytoplasm to endosomal membranes
YLL041C	SDH2	Iron-sulfur protein subunit of succinate dehydrogenase (Sdh1p, Sdh2p, Sdh3p, Sdh4p), which couples the oxidation of succinate to the transfer of electrons to ubiquinone
YLR056W	ERG3	C-5 sterol desaturase, catalyzes the introduction of a C-5(6) double bond into episterol, a precursor in ergosterol biosynthesis; mutants are viable, but cannot grow on non-fermentable carbon sources
YJL180C	ATP12	Conserved protein required for assembly of alpha and beta subunits into the F1 sector of mitochondrial F1F0 ATP synthase; mutation of human ATP12 reduces active ATP synthase levels and is associated with the disorder ATPAF2 deficiency
YKL208W	CBT1	Protein involved in 5' end processing of mitochondrial COB, 15S_rRNA, and RPM1 transcripts; may also have a role in 3' end processing of the COB pre-mRNA; displays genetic interaction with cell cycle-regulated kinase Dbf2p
YGR220C	MRPL9	Mitochondrial ribosomal protein of the large subunit
YPL013C	MRPS16	Mitochondrial ribosomal protein of the small subunit
YDR175C	RSM24	Mitochondrial ribosomal protein of the small subunit
YDR237W	MRPL7	Mitochondrial ribosomal protein of the large subunit
YDR194C	MSS116	DEAD-box protein required for efficient splicing of mitochondrial Group I and II introns; non-polar RNA helicase that also facilities strand annealing
YNL177C	MRPL22	Mitochondrial ribosomal protein of the large subunit
YBL090W	MRP21	Mitochondrial ribosomal protein of the small subunit; MRP21 exhibits genetic interactions with mutations in the COX2 and COX3 mRNA 5'-untranslated leader sequences
YBL022C	PIM1	ATP-dependent Lon protease, involved in degradation of misfolded proteins in mitochondria; required for biogenesis and maintenance of mitochondria
YBL045C	COR1	Core subunit of the ubiquinol-cytochrome c reductase complex (bc1 complex), which is a component of the mitochondrial inner membrane electron transport chain

ORF name	GENE	FUNTION
YDR231C	COX20	Mitochondrial inner membrane protein, required for proteolytic processing of Cox2p and its assembly into cytochrome c oxidase
YDR298C	ATP5	Subunit 5 of the stator stalk of mitochondrial F1F0 ATP synthase, which is an evolutionarily conserved enzyme complex required for ATP synthesis; homologous to bovine subunit OSCP (oligomycin sensitivity-conferring protein); phosphorylated
YDR322W	MRPL35	Mitochondrial ribosomal protein of the large subunit
YMR267W	PPA2	Mitochondrial inorganic pyrophosphatase, required for mitochondrial function and possibly involved in energy generation from inorganic pyrophosphate
YPL271W	ATP15	Epsilon subunit of the F1 sector of mitochondrial F1F0 ATP synthase, which is a large, evolutionarily conserved enzyme complex required for ATP synthesis; phosphorylated
YER017C	AFG3	Component, with Yta12p, of the mitochondrial inner membrane m-AAA protease that mediates degradation of misfolded or unassembled proteins and is also required for correct assembly of mitochondrial enzyme complexes
YBR179C	FZO1	Mitofusin, mitochondrial integral membrane protein involved in mitochondrial fusion and mitochondrial genome maintenance; contains N-terminal GTPase domain; targeted for destruction by cytosolic components of the ubiquitin-proteasome system
YOR200W	YOR200W	Dubious open reading frame unlikely to encode a protein, based on available experimental and comparative sequence data; partially overlaps the verified ORF MRM1/YOR201c
YOR201C	MRM1	Ribose methyltransferase that modifies a functionally critical, conserved nucleotide in mitochondrial 21S rRNA
YOR205C	GEP3	Protein of unknown function; null mutant is defective in respiration and interacts synthetically with prohibitin (phb1); the authentic, non-tagged protein is detected in purified mitochondria in high-throughput studies
YOR221C	MCT1	Predicted malonyl-CoA:ACP transferase, putative component of a type-II mitochondrial fatty acid synthase that produces intermediates for phospholipid remodeling
YGR062C	COX18	Mitochondrial integral inner membrane protein required for membrane insertion of C-terminus of Cox2p; interacts genetically and physically with Mss2p and Pnt1p; similar to S. cerevisiae Oxa1, N. crassa Oxa2p, and E. coli YidC
YMR150C	IMP1	Catalytic subunit of the mitochondrial inner membrane peptidase complex, required for maturation of mitochondrial proteins of the intermembrane space; complex contains Imp1p and Imp2p (both catalytic subunits), and Som1p
YMR256C	COX7	Subunit VII of cytochrome c oxidase, which is the terminal member of the mitochondrial inner membrane electron transport chain
YMR257C	PET111	Mitochondrial translational activator specific for the COX2 mRNA; located in the mitochondrial inner membrane

ORF name	GENE	FUNTION
YOR330C	MIP1	Catalytic subunit of the mitochondrial DNA polymerase; conserved C-terminal segment is required for the maintenance of mitochondrial genome; related to human POLG, which has been associated with mitochondrial diseases
YPL173W	MRPL40	Mitochondrial ribosomal protein of the large subunit
YPL118W	MRP51	Mitochondrial ribosomal protein of the small subunit; MRP51 exhibits genetic interactions with mutations in the COX2 and COX3 mRNA 5'-untranslated leader sequences
YPL097W	MSY1	Mitochondrial tyrosyl-tRNA synthetase
YBR251W	MRPS5	Mitochondrial ribosomal protein of the small subunit
YBR268W	MRPL37	Mitochondrial ribosomal protein of the large subunit
YDR065W	YDR065W	Protein of unknown function, required for vacuolar acidification; the authentic, non-tagged protein is detected in highly purified mitochondria in high-throughput studies
YDR079W	PET100	Chaperone that specifically facilitates the assembly of cytochrome c oxidase, integral to the mitochondrial inner membrane; interacts with a subcomplex of subunits VII, VIIa, and VIII (Cox7p, Cox9p, and Cox8p) but not with the holoenzyme
YDR375C	BCS1	Protein of the mitochondrial inner membrane that functions as an ATP-dependent chaperone, required for the incorporation of the Rip1p and Qcr10p subunits into the cytochrome bc(1) complex; member of the CDC48/PAS1/SEC18 ATPase family
YEL024W	RIP1	Ubiquinol-cytochrome-c reductase, a Rieske iron-sulfur protein of the mitochondrial cytochrome bc1 complex; transfers electrons from ubiquinol to cytochrome c1 during respiration
YEL050C	RML2	Mitochondrial ribosomal protein of the large subunit, has similarity to E. coli L2 ribosomal protein; fat21 mutant allele causes inability to utilize oleate and may interfere with activity of the Adr1p transcription factor
YER050C	RSM18	Mitochondrial ribosomal protein of the small subunit, has similarity to E. coli S18 ribosomal protein
YHL038C	CBP2	Mitochondrial protein required for splicing of the group I intron aI5 of the COB pre-mRNA, binds to the RNA to promote splicing; also involved in but not essential for splicing of the COB bI2 intron and the intron in the 21S rRNA gene
YLR201C	COQ9	Protein required for ubiquinone (coenzyme Q) biosynthesis and respiratory growth; localizes to the matrix face of the mitochondrial inner membrane in a large complex with ubiquinone biosynthetic enzymes
YKL003C	MRP17	Mitochondrial ribosomal protein of the small subunit; MRP17 exhibits genetic interactions with PET122, encoding a COX3-specific translational activator

ORF name	GENE	FUNTION
YKL170W	MRPL38	Mitochondrial ribosomal protein of the large subunit; appears as two protein spots (YmL34 and YmL38) on two-dimensional SDS gels
YKL169C	YKL169C	Dubious open reading frame unlikely to encode a protein, based on available experimental and comparative sequence data; partially overlaps the verified gene MRPL38
YJL209W	CBP1	Mitochondrial protein that interacts with the 5'-untranslated region of the COB mRNA and has a role in its stability and translation; found in a complex at the inner membrane along with Pet309p
YOR241W	MET7	Folylpolyglutamate synthetase, catalyzes extension of the glutamate chains of the folate coenzymes, required for methionine synthesis and for maintenance of mitochondrial DNA
YPL029W	SUV3	ATP-dependent RNA helicase, component of the mitochondrial degradosome along with the RNase Dss1p; the degradosome associates with the ribosome and mediates turnover of aberrant or unprocessed RNAs
YPL005W	AEP3	Peripheral mitochondrial inner membrane protein, located on the matrix face of the membrane; stabilizes the bicistronic AAP1-ATP6 mRNA encoding subunits 6 and 8 of the ATP synthase complex
YOR211C	MGM1	Mitochondrial GTPase related to dynamin, present in a complex containing Ugo1p and Fzo1p; required for normal morphology of cristae and for stability of Tim11p; homolog of human OPA1 involved in autosomal dominant optic atrophy
YDR204W	COQ4	Protein with a role in ubiquinone (Coenzyme Q) biosynthesis, possibly functioning in stabilization of Coq7p; located on the matrix face of the mitochondrial inner membrane; component of a mitochondrial ubiquinone-synthesizing complex
YLR202C	YLR202C	Dubious open reading frame unlikely to encode a protein, based on available experimental and comparative sequence data; partially overlaps the verified ORF YLR201C; ORF contains a putative intron
YLR260W	LCB5	Minor sphingoid long-chain base kinase, paralog of Lcb4p responsible for few percent of the total activity, possibly involved in synthesis of long-chain base phosphates, which function as signaling molecules
YKL002W	DID4	Class E Vps protein of the ESCRT-III complex, required for sorting of integral membrane proteins into lumenal vesicles of multivesicular bodies, and for delivery of newly synthesized vacuolar enzymes to the vacuole, involved in endocytosis
YPL065W	VPS28	Component of the ESCRT-I complex (Stp22p, Srn2p, Vps28p, and Mvb12p), which is involved in ubiquitin-dependent sorting of proteins into the endosome; conserved C-terminal domain interacts with ESCRT-III subunit Vps20p
YPL084W	BRO1	Cytoplasmic class E vacuolar protein sorting (VPS) factor that coordinates deubiquitination in the multivesicular body (MVB) pathway by recruiting Doa4p to endosomes

ORF name	GENE	FUNTION
YDR230W	YDR23 0W	Dubious open reading frame unlikely to encode a protein, based on available experimental and comparative sequence data; partially overlaps the verified gene COX20
YNL170W	YNL170 W	Dubious open reading frame unlikely to encode a functional protein, based on available experimental and comparative sequence data
YNL184C	YNL184 C	Dubious open reading frame unlikely to encode a protein, based on available experimental and comparative sequence data
YPR116W	YPR11 6W	Putative protein of unknown function; null mutation results in a decrease in plasma membrane electron transport
YOR199W	YOR19 9W	Dubious open reading frame unlikely to encode a protein, based on available experimental and comparative sequence data
YMR287C	DSS1	3'-5' exoribonuclease, component of the mitochondrial degradosome along with the ATP-dependent RNA helicase Suv3p; the degradosome associates with the ribosome and mediates turnover of aberrant or unprocessed RNAs
YMR293C	HER2	Subunit of the trimeric GatFAB AmidoTransferase(AdT) complex; involved in the formation of Q-tRNAQ; required for remodeling of ER caused by Hmg2p overexpression; similar to bacterial GatA glutamyl-tRNA amidotransferase
YLR312W-A	MRPL1 5	Mitochondrial ribosomal protein of the large subunit
YDR405W	MRP20	Mitochondrial ribosomal protein of the large subunit
YPR124W	CTR1	High-affinity copper transporter of the plasma membrane, mediates nearly all copper uptake under low copper conditions; transcriptionally induced at low copper levels and degraded at high copper levels
YLR393W	ATP10	Mitochondrial inner membrane protein required for assembly of the F0 sector of mitochondrial F1F0 ATP synthase, interacts genetically with ATP6
YBL038W	MRPL1 6	Mitochondrial ribosomal protein of the large subunit
YOL008W	COQ10	Coenzyme Q (ubiquinone) binding protein, functions in the delivery of Q6 to its proper location for electron transport during respiration; START domain protein with homologs in bacteria and eukaryotes
YMR228W	MTF1	Mitochondrial RNA polymerase specificity factor with structural similarity to S-adenosylmethionine-dependent methyltransferases and functional similarity to bacterial sigma-factors, interacts with mitochondrial core polymerase Rpo41p
YOR037W	CYC2	Mitochondrial peripheral inner membrane protein, contains a FAD cofactor in a domain exposed in the intermembrane space; exhibits redox activity in vitro; likely participates in ligation of heme to acytochromes c and c1 (Cyc1p and Cyt1p)
YOR305W	YOR30 5W	Protein of unknown function; green fluorescent protein (GFP)-fusion protein localizes to the mitochondrion; deletion confers sensitivity to 4-(N-(S-glutathionylacetyl)amino) phenylarsenoxide (GSAO); YOR305W is not an essential gene

ORF name	GENE	FUNTION
YOL033W	MSE1	Mitochondrial glutamyl-tRNA synthetase, predicted to be palmitoylated
YDR350C	ATP22	Mitochondrial inner membrane protein required for assembly of the F0 sector of mitochondrial F1F0 ATP synthase, which is a large, evolutionarily conserved enzyme complex required for ATP synthesis
YOR187W	TUF1	Mitochondrial translation elongation factor Tu; comprises both GTPase and guanine nucleotide exchange factor activities, while these activities are found in separate proteins in S. pombe and humans
YGL237C	HAP2	Subunit of the heme-activated, glucose-repressed Hap2p/3p/4p/5p CCAAT-binding complex, a transcriptional activator and global regulator of respiratory gene expression; contains sequences sufficient for both complex assembly and DNA binding
YLR417W	VPS36	Component of the ESCRT-II complex; contains the GLUE (GRAM Like Ubiquitin binding in EAP45) domain which is involved in interactions with ESCRT-I and ubiquitin-dependent sorting of proteins into the endosome
YPL002C	SNF8	Component of the ESCRT-II complex, which is involved in ubiquitin-dependent sorting of proteins into the endosome; appears to be functionally related to SNF7; involved in glucose derepression
YDR008C	YDR00 8C	Dubious open reading frame unlikely to encode a protein, based on available experimental and comparative sequence data
YHR168W	MTG2	Putative GTPase, member of the Obg family; peripheral protein of the mitochondrial inner membrane that associates with the large ribosomal subunit; required for mitochondrial translation, possibly via a role in ribosome assembly
YER061C	CEM1	Mitochondrial beta-keto-acyl synthase with possible role in fatty acid synthesis; required for mitochondrial respiration
YGR150C	CCM1	Protein required for intron removal of COB and COX1 pre-mRNAs; contains pentatricopeptide repeat (PPR) motifs; mutant is respiratory deficient and has defective plasma membrane electron transport
YCR003W	MRPL3 2	Mitochondrial ribosomal protein of the large subunit
YLR204W	QRI5	Mitochondrial inner membrane protein, required for accumulation of spliced COX1 mRNA; may have an additional role in translation of COX1 mRNA
YOL004W	SIN3	Component of the Sin3p-Rpd3p histone deacetylase complex, involved in transcriptional repression and activation of diverse processes, including mating-type switching and meiosis; involved in the maintenance of chromosomal integrity
YLR242C	ARV1	Protein functioning in transport of glycosylphosphatidylinositol intermediates into the ER lumen; required for normal intracellular sterol distribution and for sphingolipid metabolism; similar to Nup120p and C. elegans R05H5.5 protein

ORF name	GENE	FUNTION
YNL197C	WHI3	RNA binding protein that sequesters CLN3 mRNA in cytoplasmic foci; cytoplasmic retention factor for Cdc28p and associated cyclins; regulates cell fate and dose-dependently regulates the critical cell size required for passage through Start
YGL220W	FRA2	Protein involved in negative regulation of transcription of iron regulon; forms an iron independent complex with Fra2p, Grx3p, and Grx4p; null mutant fails to repress iron regulon and is sensitive to nickel
YNL213C	YNL213C	Protein of unknown function; null mutant lacks mitochondrial DNA and cannot grow on glycerol; the authentic, non-tagged protein is detected in highly purified mitochondria in high-throughput studies
YML087C	YML087C	Putative protein of unknown function, highly conserved across species and orthologous to human CYB5R4; null mutant displays reduced frequency of mitochondrial genome loss
YGR111W	YGR111W	Putative protein of unknown function; green fluorescent protein (GFP)-fusion protein localizes to both the cytoplasm and the nucleus
YMR300C	ADE4	Phosphoribosylpyrophosphate amidotransferase (PRPPAT; amidophosphoribosyltransferase), catalyzes first step of the 'de novo' purine nucleotide biosynthetic pathway
YDR295C	HDA2	Subunit of a possibly tetrameric trichostatin A-sensitive class II histone deacetylase complex containing an Hda1p homodimer and an Hda2p-Hda3p heterodimer; involved in telomere maintenance
YJL130C	URA2	Bifunctional carbamoylphosphate synthetase (CPSase)-aspartate transcarbamylase (ATCase), catalyzes the first two enzymatic steps in the de novo biosynthesis of pyrimidines; both activities are subject to feedback inhibition by UTP
YKL216W	URA1	Dihydroorotate dehydrogenase, catalyzes the fourth enzymatic step in the de novo biosynthesis of pyrimidines, converting dihydroorotic acid into orotic acid
YGL212W	VAM7	Component of the vacuole SNARE complex involved in vacuolar morphogenesis; SNAP-25 homolog; functions with a syntaxin homolog Vam3p in vacuolar protein trafficking
YGR122W	YGR122W	Probable ortholog of A. nidulans PalC, which is involved in pH regulation and binds to the ESCRT-III complex; null mutant does not properly process Rim101p and has decreased resistance to rapamycin; GFP-fusion protein is cytoplasmic
YPR149W	NCE102	Protein of unknown function; contains transmembrane domains; involved in secretion of proteins that lack classical secretory signal sequences; component of the detergent-insoluble glycolipid-enriched complexes
YOR030W	DFG16	Probable multiple transmembrane protein, involved in diploid invasive and pseudohyphal growth upon nitrogen starvation; required for accumulation of processed Rim101p

ORF name	GENE	FUNTION
YNL160W	YGP1	Cell wall-related secretory glycoprotein; induced by nutrient deprivation-associated growth arrest and upon entry into stationary phase; may be involved in adaptation prior to stationary phase entry; has similarity to Sps100p
YBL012C	YBL012C	Dubious open reading frame unlikely to encode a protein, based on available experimental and comparative sequence data
YBL044W	YBL0044	Putative protein of unknown function; YBL044W is not an essential protein
YGL234W	ADE5	Bifunctional enzyme of the 'de novo' purine nucleotide biosynthetic pathway, contains aminoimidazole ribotide synthetase and glycinamide ribotide synthetase activities
YMR184W	ADD37	Protein of unknown function involved in ER-associated protein degradation; green fluorescent protein (GFP)-fusion protein localizes to the cytoplasm and is induced in response to the DNA-damaging agent MMS; YMR184W is not an essential gene
YMR244C-A	YMR244C-A	Putative protein of unknown function; green fluorescent protein (GFP)-fusion protein localizes to both the cytoplasm and nucleus and is induced in response to the DNA-damaging agent MMS; YMR244C-A is not an essential gene
YGR109C	CLB6	B-type cyclin involved in DNA replication during S phase; activates Cdc28p to promote initiation of DNA synthesis; functions in formation of mitotic spindles along with Clb3p and Clb4p; most abundant during late G1
YPL132W	COX11	Mitochondrial inner membrane protein required for delivery of copper to the Cox1p subunit of cytochrome c oxidase; association with mitochondrial ribosomes suggests that copper delivery may occur during translation of Cox1p
YJL102W	MEF2	Mitochondrial elongation factor involved in translational elongation
YJL096W	MRPL49	Mitochondrial ribosomal protein of the large subunit
YAL047C	SPC72	Component of the cytoplasmic Tub4p (gamma-tubulin) complex, binds spindle pole bodies and links them to microtubules; has roles in astral microtubule formation and stabilization
YAL012W	CYS3	Cystathionine gamma-lyase, catalyzes one of the two reactions involved in the transsulfuration pathway that yields cysteine from homocysteine with the intermediary formation of cystathionine
YJR144W	MGM101	Protein involved in mitochondrial genome maintenance; component of the mitochondrial nucleoid, required for the repair of oxidative mtDNA damage
YMR089C	YTA12	Component, with Afg3p, of the mitochondrial inner membrane m-AAA protease that mediates degradation of misfolded or unassembled proteins and is also required for correct assembly of mitochondrial enzyme complexes

ORF name	GENE	FUNTION
YBR026C	ETR1	2-enoyl thioester reductase, member of the medium chain dehydrogenase/reductase family; localized to in mitochondria, where it has a probable role in fatty acid synthesis
YCR024C	SLM5	Mitochondrial asparaginyl-tRNA synthetase
YNR036C	MRPS12	Mitochondrial protein; may interact with ribosomes based on co-purification experiments; similar to E. coli and human mitochondrial S12 ribosomal proteins
YNL005C	MRP2	Mitochondrial ribosomal protein of the large subunit
YCR071C	IMG2	Mitochondrial ribosomal protein of the small subunit
YNR037C	RSM19	Mitochondrial ribosomal protein of the small subunit, has similarity to E. coli S19 ribosomal protein
YPR047W	MSF1	Mitochondrial phenylalanyl-tRNA synthetase, active as a monomer, unlike the cytoplasmic subunit which is active as a dimer complexed to a beta subunit dimer; similar to the alpha subunit of E. coli phenylalanyl-tRNA synthetase
YPR100W	MRPL51	Mitochondrial ribosomal protein of the large subunit
YLL018C-A	COX19	Protein required for cytochrome c oxidase assembly, located in the cytosol and mitochondrial intermembrane space; putative copper metallochaperone that delivers copper to cytochrome c oxidase
YMR158W	MRPS8	Mitochondrial ribosomal protein of the small subunit
YDL107W	MSS2	Peripherally bound inner membrane protein of the mitochondrial matrix involved in membrane insertion of C-terminus of Cox2p, interacts genetically and physically with Cox18p
YJR113C	RSM7	Mitochondrial ribosomal protein of the small subunit, has similarity to E. coli S7 ribosomal protein
YJL023C	PET130	Protein required for respiratory growth; the authentic, non-tagged protein is detected in highly purified mitochondria in high-throughput studies
YNR041C	COQ2	Para hydroxybenzoate: polyprenyl transferase, catalyzes the second step in ubiquinone (coenzyme Q) biosynthesis
YBL100C	YBL100C	Dubious open reading frame unlikely to encode a protein, based on available experimental and comparative sequence data; almost completely overlaps the 5' end of ATP1
YJL063C	MRPL8	Mitochondrial ribosomal protein of the large subunit
YJR077C	MIR1	Mitochondrial phosphate carrier, imports inorganic phosphate into mitochondria; functionally redundant with Pic2p but more abundant than Pic2p under normal conditions; phosphorylated

ORF name	GENE	FUNTION
YBR037C	SCO1	Copper-binding protein of the mitochondrial inner membrane, required for cytochrome c oxidase activity and respiration; may function to deliver copper to cytochrome c oxidase; has similarity to thioredoxins
YNL081C	SWS2	Putative mitochondrial ribosomal protein of the small subunit, has similarity to E. coli S13 ribosomal protein; participates in controlling sporulation efficiency
YBR003W	COQ1	Hexaprenyl pyrophosphate synthetase, catalyzes the first step in ubiquinone (coenzyme Q) biosynthesis
YDL146W	LDB17	Protein involved in the regulation of endocytosis; transiently recruited to actin cortical patches in a SLA1-dependent manner after late coat component assembly; GFP-fusion protein localizes to the periphery, cytoplasm, bud, and bud neck
YDR042C	YDR04 2C	Putative protein of unknown function; expression is increased in ssu72-ts69 mutant
YBL099W	ATP1	Alpha subunit of the F1 sector of mitochondrial F1F0 ATP synthase, which is a large, evolutionarily conserved enzyme complex required for ATP synthesis; phosphorylated
YJR120W	YJR120 W	Protein of unknown function; essential for growth under anaerobic conditions; mutation causes decreased expression of ATP2, impaired respiration, defective sterol uptake, and altered levels/localization of ABC transporters Aus1p and Pdr11p
YJR122W	IBA57	Mitochondrial matrix protein involved in the incorporation of iron-sulfur clusters into mitochondrial aconitase-type proteins; activates the radical-SAM family members Bio2p and Lip5p; interacts with Ccr4p in the two-hybrid system
YDL198C	GGC1	Mitochondrial GTP/GDP transporter, essential for mitochondrial genome maintenance; has a role in mitochondrial iron transport; member of the mitochondrial carrier family
YNR042W	YNR04 2W	Dubious open reading frame unlikely to encode a protein, based on available experimental and comparative sequence data; completely overlaps verified gene COQ2
YNR020C	ATP23	Putative metalloprotease of the mitochondrial inner membrane, required for processing of Atp6p; has an additional role in assembly of the F0 sector of the F1F0 ATP synthase complex
YLR038C	COX12	Subunit VIb of cytochrome c oxidase, which is the terminal member of the mitochondrial inner membrane electron transport chain; required for assembly of cytochrome c oxidase but not required for activity after assembly; phosphorylated
YBR282W	MRPL2 7	Mitochondrial ribosomal protein of the large subunit
YNL064C	YDJ1	Protein chaperone involved in regulation of the HSP90 and HSP70 functions; involved in protein translocation across membranes; member of the DnaJ family

ORF name	GENE	FUNTION
YNR001C	CIT1	Citrate synthase, catalyzes the condensation of acetyl coenzyme A and oxaloacetate to form citrate; the rate-limiting enzyme of the TCA cycle; nuclear encoded mitochondrial protein
YJR048W	CYC1	Cytochrome c, isoform 1; electron carrier of the mitochondrial intermembrane space that transfers electrons from ubiquinone-cytochrome c oxidoreductase to cytochrome c oxidase during cellular respiration
YJR080C	AIM24	Protein of unknown function; the authentic, non-tagged protein is detected in purified mitochondria in high-throughput studies; null mutant displays reduced respiratory growth and elevated frequency of mitochondrial genome loss
YNL037C	IDH1	Subunit of mitochondrial NAD(+)-dependent isocitrate dehydrogenase, which catalyzes the oxidation of isocitrate to alpha-ketoglutarate in the TCA cycle
YBR044C	TCM62	Protein involved in the assembly of the mitochondrial succinate dehydrogenase complex; putative chaperone
YDL181W	INH1	Protein that inhibits ATP hydrolysis by the F1F0-ATP synthase; inhibitory function is enhanced by stabilizing proteins Stf1p and Stf2p; has similarity to Stf1p; has a calmodulin-binding motif and binds calmodulin in vitro
YNL117W	MLS1	Malate synthase, enzyme of the glyoxylate cycle, involved in utilization of non-fermentable carbon sources; expression is subject to carbon catabolite repression; localizes in peroxisomes during growth in oleic acid medium
YDL192W	ARF1	ADP-ribosylation factor, GTPase of the Ras superfamily involved in regulation of coated vesicle formation in intracellular trafficking within the Golgi; functionally interchangeable with Arf2p
YJR102C	VPS25	Component of the ESCRT-II complex, which is involved in ubiquitin-dependent sorting of proteins into the endosome
YNR010W	CSE2	Subunit of the RNA polymerase II mediator complex; associates with core polymerase subunits to form the RNA polymerase II holoenzyme; component of the Med9/10 module; required for regulation of RNA polymerase II activity
YLR420W	URA4	Dihydroorotase, catalyzes the third enzymatic step in the de novo biosynthesis of pyrimidines, converting carbamoyl-L-aspartate into dihydroorotate
